# Viral DNA replication: new insights and discoveries from large scale computational analysis

**DOI:** 10.1186/1471-2105-16-S3-A2

**Published:** 2015-02-13

**Authors:** Darius Kazlauskas, Česlovas Venclovas

**Affiliations:** 1 Institute of Biotechnology, Vilnius University, Lithuania

## Background

The ability to replicate is essential for all living entities. Duplication of genetic information is carried out by replication proteins. DNA replication has been well studied in T7, T4 phages and herpes viruses; however, the information about replication mechanisms from other groups of viruses is either scarce or missing altogether. Double-stranded (ds) DNA viruses infect cells from all domains of life, they evolve fast and are very diverse. Their genome size varies from 5 to 2,500 kbp.

## Results and conclusions

To better understand viral DNA replication, we identified replication proteins in dsDNA viruses using current state-of-the-art homology detection methods. Over 150,000 proteins from 1,574 genomes were analyzed. We found that the composition of replication machinery depends on the virus genome size. Small viruses (<40 kbp) use protein-primed DNA replication or rely on replication proteins from the host. Large viruses (>140 kbp) have their own RNA-primed replication apparatus often supplemented with processivity factors and DNA topoisomerases to increase replication speed and efficiency. This insight led us to a search for „missing“ replication components in large genomes and resulted in the discovery of single-stranded DNA binding (SSB) proteins in larger eukaryotic viruses. Surprisingly these proteins turned out to be homologs of SSB proteins previously thought to be specific for T7-like phages. Additionally with the analysis of the herpes viral helicase-primase complex we found that one of its components, UL8, is a highly diverged inactivated B-family DNA polymerase.

